# Supplementary material for: Utilization of accessible resources in the fabrication of an affordable, portable, high-resolution, 3D printed, digital microscope for Philippine diagnostic applications
Source: PLOS Glob Public Health. 2023 Nov 21;3(11):e0002070. doi: 10.1371/journal.pgph.0002070 (PMC10662710; doi:10.1371/journal.pgph.0002070)
Supplement: Attachment — Submitted filename: Response to Reviewers PLOS Global Public Health 23 May 2023.docx [file pgph.0002070.s001.docx]

25 May 2023

Senjuti Saha

Guest Editor

PLOS Global Public Health

Dear Editor,

Thank you for your patience and for sending us the comments from our peer reviewers. As instructed, we have prepared our responses to each point raised by them below in table format.

Table 1 is for the general editorial comments, table 2 is for reviewer 1 and table 3 is for reviewer 2.

We hope our responses are satisfactory and we are happy to provide more clarifications and responses with the contents of our paper.

**Table 1**. Editor comments and responses

| **Editor Comments** | **Response** | **Reference in Revised Manuscript** |
| --- | --- | --- |
| Please send a completed 'Competing Interests' statement, including any COIs declared by your co-authors. If you have no competing interests to declare, please state "The authors have declared that no competing interests exist". Otherwise please declare all competing interests beginning with the statement "I have read the journal's policy and the authors of this manuscript have the following competing interests:" | Dear Editor,  Thank you for your comment. We have revised the competing interest and financial disclosure statements in the manuscript.  Dr. Jeremie De Guzman has declared his commercial interests while Dr. Mark Kristan Cabello has declared no conflicts. | Please see lines 491 to 495 page 29 |
| Please amend your detailed Financial Disclosure statement. This is published with the article. It must therefore be completed in full sentences and contain the exact wording you wish to be published.  a. State the initials, alongside each funding source, of each author to receive each grant.  b. State what role the funders took in the study. If the funders had no role in your study, please state: “The funders had no role in study design, data collection and analysis, decision to publish, or preparation of the manuscript.”  c. If any authors received a salary from any of your funders, please state which authors and which funders. | Dear Editor,  Thank you. We have added a revised conflict of interest and financial disclosure statement in the manuscript. | Please see lines 484 to 490 pages 28 - 29 |

**Table 2. Reviewer 1 comments and responses**

| **Reviewer 1 Comment** | **Response** | **Reference in revised manuscript** |
| --- | --- | --- |
| 1. Reviewer #1: Material and Methods:   Lines 84 – 91: If authors decided to mention the list of materials, they should share a full description of the list instead of listing few parts. For readers, it can be convenient to know which components were available in the Philippines and which were imported or replaced by others. | Dear Reviewer,  Thank you for your comments.  Lines 84-91: We had included a table to summarize and describe all parts and components our team assembled and used for this study. We had added text to indicate where we had purchased these and remarks on their function. | Please see Table 1 line 116 page 6 - 9 |
| Lines 92, 93: Authors should explain why they used different CAD software. Authors should highlight Fusion 360 has only a 30-days trial for free use and Sketchup Pro is a paid software. These lines are not consistent with the abstract and conclusions. | Dear Reviewer,  Thank you for this comment. For lines 92,93: To our knowledge, there is a free Fusion 360 license for academic institutions, educators, students, and hobbyists. At present, these could be renewed indefinitely to keep using the core features of the software. A trial license of Sketchup Pro 2021 was used initially to help draw some of the components, but these were eventually migrated to Fusion 360 where all subsequent modifications and modeling was completed. | Please see lines 94 to 98 page 5 |
| Line 95, 96: Due to this journal is not an engineering journal, authors should be explained more about the files, how they were generated, and in which stages they were relevant for manufacturing. | Dear Reviewer,  Thank you for this comment. Lines 95,96: The OFM, as an existing open source hardware project, made all 3d printed component models, software, and build instructions for free at their website at openflexure.org. Our team adopted their existing files and modified them to build a local version. These materials are distributed under the CERN open hardware license. At present, the team manufactured the high-optical grade version as we were interested in eventually testing the unit we fabricated for different clinical microscopy scenarios. | Please see lines 90 to 83 page 5 |
| Lines 124, 125: What does “temperature was optimized” mean? Were the 3D printer or extrusion system modified for this purpose? 3D Printer temperature can be set easily using the software or hardware interface. | Dear Reviewer,  Thank you for this comment. For Lines 124-125: Temperature optimization in this instance refers to the temperature of the 3d printer nozzle and build plate. Indeed, the temperature can be set easily, but finding the correct temperature settings is needed for optimizing build quality, durability, and fidelity to the designed components. We found it was necessary to do this because the ambient conditions of the Philippines (environmental temperature and relative humidity) affects build quality and we had to experiment on optimal settings to produce the most consistent print that we could that would reducing non-adhesions, warping, and deformities. We had included similar text in the article reflecting our response here. | Please see lines 99 to 104 page 5 |
| Reviewer 1 - Results and Discussion:  Fig 1: How was the Open Flexure Microscope (OFM) used? A keyboard, mouse, and a screen were connected? How were the Arduino and Raspberry Pi connected each other? Please, share more images from the setup and it is recommend a short video to demonstrate slide scanning capability of the OFM. | Dear Reviewer,  Thank you for your comment. For figure 1 we had included the setup we had used as you suggested. For the arduino and raspberry pi these were connected using wires and placed in dedicated fittings in the base of the Microscope. While a video would be ideal, we plan to produce this at a later date. | Please see Figure 1 line 56 page 3  Figure 2 line 171 page 12 |
| Lines 124, 125: What does “temperature was optimized” mean? Were the 3D printer or extrusion system modified for this purpose? 3D Printer temperature can be set easily using the software or hardware interface. | Dear Reviewer,  Thank you for your comment. For lines 124-125 please see our response above. | Please see lines 99 to 104 page 5 |
| Lines 127-129: What does “trials” mean? To print several times or perform mechanical tests? | Dear Reviewer,  Thank you for your comment. For lines 127-129: We had multiple prints to test the printing parameters. This is what we meant by trial print. Each of these printed parts were subjected to daily use and we qualitatively evaluated its durability. We had conducted no formal mechanical tests and instead when a part breaks down we printed a new version and served as our proxy for durability when a component withstood regular use. | Please see lines 176 to 180 page 12 |
| Fig 2: All the images should have a scale reference for showing the OFM optics capabilities. | Dear Reviewer,  Thank you for your comment. For Fig 2. We agree and have included a scale reference for each image. We have also revised this into figure 3. | Please see Figure 3, lines 225 to 233, page 14 - 15 |
| Lines 188, 189: Raspberry Pi storing capacity does not depend on computer capacity, but it depends on SD cards or external storages. | Dear Reviewer,  Thank you for your comment. For Lines 188, 189: To clarify, the 8GB version refers to the RAM capacity of the Rpi 4B and not the hard drive storage. We had opted for this variant model to ensure better performance and responsiveness of the computer. The RPi 4B also has greater onboard storage capacity which can be expanded by MicroSD cards. We have removed these lines from the discussion to focus instead on the cost implications and why our unit is more expensive than what was quoted in literature. | Not applicable. We have removed lines 188 to 189 in our latest revised manuscript |
| Lines 190, 191: Why were not the lens of the camera or the web cam version of the OFM used in this study? | Dear Reviewer,  Thank you for your comment. For Lines 190, 191, there are two versions of the OFM design, the webcam ONLY version, and the hybrid high-optics model. The former is only up to a total of 100x digital magnification, while high optics versions can go up to 1000x digital-analog hybrid magnification. We chose the high optics model for clinical or high magnification purposes. The high-optics model already incorporates the web-cam sensor alongside standard RMS threaded objective lenses. | Not applicable |
| Line 195, Table 1: It could be useful see a detailed list of costs per components. | Dear Reviewer,  Thank you for your comment. For Line 195, we had incorporated the detailed parts per category and listed the individual costs in and revised this into Table 4. | Please see table 4 line 252 pages 15 - 17 |
| Line 224, 225: In this study, it was used only an open-source design but the manufacturing tools such as CAD software and 3D printer were proprietary technologies. | Dear Reviewer,  Thank you for your comment. For Lines 224, 225 We recognize that for our study we purchased a desktop commercial 3d printer and used Autodesk Fusion 360 (which is mainly a commercial CAD software). However, desktop fusion deposited modeling (FDM) 3d printers have recently been more affordable and available in the Philippine market. We were able to purchase a unit for approximately $372.3. For the CAD software we already mentioned how Fusion 360 has a feature-limited license for free for academics and educators. | Please see lines 94 to 98 page 5  Please see also lines 291 to 296 page 20 |
| Lines 231, 232: This is speculation. No tests or comparison were documented in this article. Only reports from end-users were shared. | Dear Reviewer,  Thank you for your comment. For Lines 231, 232: We had included one of our characterization experiments in the paper which calculated the resolution of our OFM unit compared to an LED light microscope with the image captured by a smartphone (an Iphone 12 camera with no attachments). We had written the results of our calculations in table 3. | Please see table 3 line 209 to 212 page 13 - 14 |
| Lines 233-235: It could be useful to have model references and prices from the Philippine market. | Dear Reviewer,  Thank you for your comment. For Lines 233-235: We have compiled some prices from online marketplaces where microscopes could be purchased that have similar features to our OFM unit in table 5. Although this is not an exhaustive list, it does provide some comparison about our LFM cost compared to commercial units. | Please see table 5 line 262 page 17 to 18 |
| Lines 305, 306: Cite reference for this sentence. | Dear Reviewer,  Thank you for your comment. For lines 305 to 306, we would like to clarify that we have not found any other study that has integrated the use of a smartphone with the OFM as its main user interface instead of a laptop or a desktop computer. We plan to investigate this in a future iteration of the OFM unit. The interest in this statement is to see the capacity of the OFM in adapting to existing equipment within health facilities. For example, physicians would more likely have smartphones rather than a computer that they already have in their possession. We have removed this sentence because we have yet to implement this feature in our current iteration model and will be the subject of another project. | Not applicable as we have removed this sentence. |
| Conclusions and future directions  Lines 325-327: How was tested these capabilities? Only visual examination? end-users were experts? | Dear Reviewer,  Thank you for this comment. For the capability of the OFM in taking images, we had taken these from pathology teaching slides to qualitatively assess the images taken by the unit. The examination of the samples were only visual as these slides have already been processed previously with a known interpretation. Furthermore, one of the authors, Dr. Jeremie, is a general physician with training in microscopy and used this to identify some pathologic features in the slide with the help of a pathology manual.  Future work that we intend to conduct would involve doing a full diagnostic accuracy study to assess its performance in simulated clinical use cases.  The other test we had for the OFM was to calculate its image resolution compared with an LED light microscope where the image was captured using a mobile phone. We had used ImageJ, an image-processing software for optical and computational instrumentation, to produce these resolution calculations. | Please see lines 207 to 212 pages 13 - 14  Please see lines 144 to 151 pages 10 - 11  Please see lines 214 to 243 pages 14 - 15 |

**Table 2**. Reviewer 2 comments and responses

| **Comment** | **Response** | **Revision reference** |
| --- | --- | --- |
| Abstract: This section is well written and exhaustively discussed. However I am  of the opinion that the sections on the “future work”, lines 28 to 32 be excluded  from the abstract as it majorly focuses on the recommendation for future studies. I suggest that the authors to make the abstract as brief but exhaustive as possible, and allow it to end with the significance of their findings. | Dear Reviewer,  Thank you for this comment, we have removed these lines and made minor changes to reflect the significance in our end statement. | Please see abstract lines 10 to 28 pages 1 - 2 |
| Rationale for building the Openflexture microscope: The authors have  reasonably discussed the rationale as to why they chose Openflexure  microscope over other available design.  I would like to point out that the sentence in lines 63 to 67 is rather long  and it makes one lose the gist in it. | Dear Reviewer,  For lines 63 to 67, we had revised it to divide the sentence into more understandable segments. | Please see lines 68 to 73 pages 3 - 4. |
| Materials and Methods: Generally well written and quite informative, besides a  few grammatical errors that needs to be rechecked:  Line 84 to 85: (….was used for easily fabricating 85 3D printed  parts…) instead the authors should consider use of correct tense. | Dear Reviewer,  Thank you for this. We have corrected our grammar to the correct tense. | Please see lines 94 to 95 page 5 |
| It will be important for the authors to state their experimental design and  provide data on the same in order to understand whether OFM results  are reproducible and how it compares to conventional microscopy. | Dear Reviewer,  Thank you for this comment. We had added additional sections to clarify what our team did to characterize and do initial assessment of the performance of the OFM.  I would also like to clarify that we have yet to do a full diagnostic performance study with the OFM as our goals with this current phase of the project is to produce the OFM, determine the costs of fabrication within the Philippine context, and characterize its imaging capabilities. We used the images obtained by the OFM and conventional microscopes to identify pathologic features from teaching slides available at our university.  Our goal for the next part of the project is to compare an upgraded OFM to conventional microscopes to determine its diagnostic performance. Our outputs would hopefully result in the diagnostic agreement, sensitivity, specificity, negative predictive value, and positive predictive value.  To our knowledge publications discussing the OFM have used it more for research settings, or for pilot studies assessing its feasibility on certain use cases. Malaria is the only one we found from Knapper et al., 2022 where its potential in public health and clinical services was mentioned. Hence, we would want to assess its performance for neglected tropical diseases, tuberculosis, and other basic diagnostics including blood counts, urinalysis and fecalysis. All of which are relevant conditions and diagnostics for primary care.  Within the article we have added text describing the experiments we had done. These included the image assessment, the image resolution calculation, and costing we had made for the OFM. | Please see lines 134 to 167 pages 10 to 11. |
| Results and discussions:  I am of the opinion that lines 122 all the way to 133, which talks more  on the fabrication process and not the actual results, is misplaced and  may fit well in materials and methods section. | Dear Reviewer,  Thank you for pointing this out. We have moved this section to the materials and methods. | Please see lines 169 to 199 pages 11 - 13 |
| The authors should consider restructuring lines 181 to 185 which is seen  as giving free marketing campaign to online stores, and is not within the  aims and objectives of the study. In line to this, lines 181 to 191 may not  fit well with the results and discussion section as it is more of “what the  authors did”, I suggest they restructure it to bring out the results of the  study and possibly the discussion where they compare their findings  with similar work elsewhere bringing out their key findings. | Dear Reviewer,  We have edited lines 181 to 185 to deemphasize online stores and purchasing.  lines 181 to 191 to emphasize the costs we incurred with fabricating the OFM unit. We have added an additional table 5 to expand on the costs of other microscopes available in the Market.  Furthermore, we have added additional sections to discuss the image resolution performance of our OFM unit and our findings examining pathology teaching slides captured using the same unit. | Please see lines 245 to 269 pages 16 - 19  Please see also lines 207 to 243 pages 13 - 15 |
| Line 192: It is not quite clear to decipher the mind of the author, when  they say that “3D printed plastic microscope parts took 52 hours to be  printed…” do they mean the whole procedure of setting up took 52  hours or just printing the microscopy results? | Dear Reviewer,  Thank you for this comment. To clarify, this line is the total time it took for our 3d printer to fabricate all the microscope parts to assemble the OFM. We have listed all the 3d printed components in table 2 for reference.  We also did not have a printed report for the microscopy results. The images were directly taken by the OFM and then interpreted and annotated within the OFM unit as part of its software interface. For the conventional microscopes, after we were able to focus the slides of interest, we simply took a picture using a smartphone. | Not applicable |
| Besides designing and coming up with a novel microscope, authors  should have compared the images from OFM to the available  conventional microscope and other microscopic technologies at the same  magnifications. Through this, purposive comparisons of the image  resolution and clarity could authoritatively be brought out. | Dear Reviewer,  Thank you for this comment. We have added some of our additional characterization experiments to compare the image resolution of our OFM unit with a conventional microscope that was readily available for use in our settings. Our initial image acquisition and qualitative assessment was only meant to demonstrate its utility, and possible applicability to frontline healthcare facilities.  We recognize that we should have made more comparisons, however this was beyond the scope of this research project.  Our plan is to conduct diagnostic accuracy studies between conventional microscopes used in the field alongside an improved OFM design for public health relevant conditions and use cases. With this future work, we hope we will have more authoritative data to demonstrate its performance. | Please see also lines 207 to 243 pages 13 - 15 |
| The authors have highlighted the disadvantages of the conventional  microscopes that require trained personnel. It would also be important to  bring out the challenges, pros and cons facing the Openflexture  microscope. Therefore, it will imperative for the authors to discuss these  factors in detail as they compare it with other microscopic technologies | Dear Reviewer,  We agree that we should discuss the limitations of the OpenFlexure microscope as well as its challenges compared to other microscopes. We have added text discussing this. | Please see lines 392 to 425 pages 24 - 26 |
| Lines 249 to 251 are more of recommendation for future studies and do  not sit well in this section. | Dear Reviewer,  Thank you for this. We have revised the conclusions and discussions and have integrated these in lines 463 - 465 and lines 468 to 469 | Please see lines 462 - 464 and lines 465 to 468 pages 27 - 28 |
| In line 262 to 263, the authors points out that, evidence suggests that  telemedicine may also apply, telepathology may be cost effective. It will  be important to compare this point with work done elsewhere and to cite  a few articles to give credence to this statement. | Dear Reviewer,  Thank you for this comment. We have added a few articles to highlight the attractiveness and potential cost savings of telepathology usage.  Considering the OFM already is internet-enabled at the outset, testing its capabilities for telepathology and its diagnostic performance vis-a-vis its costs to implement are subsequent research areas we intend to explore. | Please see lines 337 to 346 pages 21 - 22 |
| I would like to agree with the authors that the idea and the technology is  quite novel, and the cost of fabrication (322 USD) is quite affordable.  However, when they say the cost of acquiring and installing one unit is  about 835,000 USD (line 274-275), is that quite high and far reaching to  most facilities in resource limited countries, like Philippines? Having in  mind that the cost of acquiring a conventional light microscope that can  have a magnification of up to x1000 is roughly between 100 USD to  1,330 USD. What will be the rationale of a facility going for a  microscope that will cost 835,000 USD, yet they can acquire up to about  627 olympus light microscope at the same price. Kindly elaborate more  on the cost implications. | Dear Reviewer,  We apologize for the confusion. We had calculated the $835,000 more as a theoretical total cost to build 2,593 OFM units to supply each rural health unit in the Philippines with one OFM unit. We have removed this line to avoid any confusion. Instead we have provided more detailed discussion on the cost drivers in fabricating the OFM. In addition, we have provided for a table to compare the costs and features of the OFM we had made compared to commercially available ones.  We have also provided some discussion about the advantages and disadvantages of the OFM. We acknowledge that at present, its features may not be enough to encourage wholesale adoption, but there is potential for it to become more attractive to Philippine health facilities given more modifications and improvements. | Please see lines 253 to 261 page17  Please see table 4 line 252 page 15 - 17  Please see table 6, line 416 page 25 |
| Lines 277 to 300: the authors have introduce the concept of mobile  phone microscopy so well, but digressed out of the current context to  talk about something they are yet to test and, or dont have data to  support it. As such, it is not clear how OFM is being integrated to  mobile microscopy and how this has facilitated its acceptability within  the health facilities. | Dear Reviewer,  Thank you for this comment. We have removed this part of the discussion because we have yet to develop this feature.  Our intent with future work is to explore ways in which we could integrate mobile phones with the OFM. Given that mobile phones already have screens with touch sensors. Both of these standard features of today’s smartphones could be integrated with the OFM as its display and control mechanism for moving and annotating slides.  In this way, it can decrease the needed infrastructure requirements so facilities would no longer need to buy dedicated laptops, desktops, or monitors to use with the OFM if they already have mobile phones.  As it stands the OFM does need these accessories to function and we have added text in the limitations of the unit explaining this.  We have yet to explore in detail if the OFM would be more attractive for health facilities to use. But we believe that by incorporating end-user inputs into the design we could create an iteration of the OFM that would be easier to adopt for local health facilities. | Please see lines 417 to 425, pages 25 - 26 |
| Conclusion: The conclusion is well written, but quite brief. It has only  concentrated mostly on what needs to be done in the future rather than the  summary of the study findings. Therefore, the authors of the current study need  to bring out what was the finding of the study, its applicability and practicability  to public health facilities. | Dear Reviewer,  Thank you for this. We have revised the conclusion to summarize our major findings as well as challenges for the OFM in implementing it for use in public health facilities. | Please see lines 427 to 477 pages 26 - 28 |
| NB: The rationale of designing and fabrication of OFM using locally  sourced materials was to have a cheaper and easily accessed microscope.  Through out the document, authors have OFM as a cheap microscope.  However, in their conclusion (line 327 to 328), the study argues that the  cost of acquisition of OFM is quite higher than quoted in the literature. | Dear Reviewer,  Thank you for this comment. We acknowledge that in our costing and conclusions that the unit we fabricated appears to be more expensive than what was quoted in literature. However, we had included discussion on why this might be the case. The original designers quoted the $200 price only covering parts to build the unit, but did not include other incidental costs. Also, since the time of the publication, there might have been increases in the price of the components, taxes, and other factors that made the price when we fabricated it higher. We have added some discussion talking about this finding.  Furthermore, we added table 5, which compares the OFM unit we fabricated with other commercially available microscopes. Among microscopes with a digital camera attached, our unit has the lowest cost with even more advanced features. | Please see table 4 and lines 252 to 261 pages 15 - 17  Please see table 5 line 262 pages 17 - 18 |
| Thus, the study discussion and conclusions is a bit lacking in comparing  and contrasting the findings of the OFM to other conventional  microscopy. | Dear Reviewer,  Thank you for this comment. We realized that we had not given more data to demonstrate how our OFM was different to conventional microscopy. We have added additional figures and descriptions to directly compare the image outputs of the OFM and conventional microscopy. We had added an image resolution calculation between the two modalities. Although for the latter we had used a mobile phone to capture the images as we did not have a unit with in-built digital camera available to us. We have also included tables to compare costs and another to summarize the advantages and disadvantages of each modality from our perspective. | Please see lines 134 to 167 pages 10 - 11  Please see lines 207 to 212 pages 13 - 14  Please see lines 245 to 261 pages 15 - 17  Please see table 4, table 5 and table 6 |

Very sincerely yours,

Jeremie E. De Guzman MD., MBA., MSc
