# Supplementary material for: Utilization of accessible resources in the fabrication of an affordable, portable, high-resolution, 3D printed, digital microscope for Philippine diagnostic applications
Source: PLOS Glob Public Health. 2023 Nov 21;3(11):e0002070. doi: 10.1371/journal.pgph.0002070 (PMC10662710; doi:10.1371/journal.pgph.0002070)
Supplement: Attachment — Submitted filename: Response to Reviewers PLOS Global Public Health 27 Aug 2023.docx [file pgph.0002070.s002.docx]

27 August 2023

Senjuti Saha

Guest Editor

PLOS Global Public Health

Dear Editor,

Thank you for your patience and for sending us the comments from our peer reviewers. As instructed, we have prepared our responses to each point raised by them below in table format.

**Table 1**. **Reviewer comments and responses by authors**

| **Reviewer 1 Comment** | **Response** | **Reference in revised manuscript** |
| --- | --- | --- |
| - To review the manuscript and correct the typos in the text and table content. | Dear Reviewer,  Thank you for pointing this out. We have continued to correct the typos and grammatical errors and hope all of them have been corrected in this submission. | N/A |
| - To correct scale in images D and H from Figure 3. | Dear Reviewer,  We have corrected the reference scale in Figure 3, image D and H and is attached as figure 3_v4 | Please see Figure 3_v4 |
| - To explain why an inverted microscope (OFM) was built to compare it with an upright microscope (conventional light microscope). There is an upright microscope version of the OFM: <https://build.openflexure.org/openflexure-microscope/v7.0.0-alpha2/upright-microscope.html> <https://openflexure.discourse.group/t/freshly-assembled-upright-microscope-v7-0-0-beta1/1151> | Dear Reviewer,  Thank you for this comment. The upright microscope design of the OFM was designed for the low-optics version.  Because of this, we decided to use the inverted high-optics version because we were considering the need for 1000x magnification when used for clinical laboratory settings. | n/a |
| Abstract:  - To mention "TB" means "tuberculosis" and not after. | Dear Reviewer,  Thank you for pointing this out. We had included the full name tuberculosis before using its acronym of TB | Abstract section line 11, page 1 |
| - To verify if the OFM is a prototype or a final product. The term "prototype" suggests that this microscope is still far from being used in health facilities. | Dear Reviewer,  You are correct that the OFM is still a prototype that is not yet ready for wide scale deployment in health facilities.  We have added text stating this in the discussion section.  We also mentioned in our conclusion that future work is dedicated to advancing our prototype towards implementation in health facilities. That would require redesigning the OFM based on inputs from different stakeholders, contextual factors, and use case scenarios. | Discussion section  Lines 394 to 395, page 24  Conclusion section lines 476 to 483, page 29 |
| Materials and Method:  - To improve the description of the terms "OpenFlexure Operating System" and "OpenFlexure Connect" (GUI). In this section, the term "software" is used for both solutions but they have different purposes. | Dear Reviewer,  Please see table 1, row 5. We had added remarks to describe the installed raspbian-openflexure software package and operating system.  Furthermore, we had added additional text to describe the software package in the body. | Table 1, row 6 |
| - To share the sensor resolution of the Raspberry Pi camera and smartphone. | Dear Reviewer,  We have added the resolution of the Rpi camera v2 and the iPhone 12 smartphone we used to determine the resolution of the OFM system we fabricated.  The Rpi camera v2 has 8 megapixels resolution and the iPhone 12 has 12 megapixels of resolution. | Table 1 row 17  Materials and methods line 143, page 11 |
| - To share or update images where the ring for the condenser is used. | Dear Reviewer,  We have updated figure 1 to show where the ring for the condenser was used. | Please see attached figure 1_v3 |
| - If a smartphone is needed for digital microscopy in a real context, its price should be included. | Dear Reviewer,  In our present setup we used a computer and monitor with the OFM. While a smartphone could be used by facilities or other potential users, in our study we only used with a conventional microscope and not as the main interface and viewing tool with the OFM.  We feel that our setup would be the initial configuration and costed it that way.  Using a smartphone and other digital displays and control methods are approaches we plan to explore in another project with the OFM alongside likely modifications to the design. In this future project, we also will consider the costs of the different setups, including our recommended smartphone types and specs once we have been able to study them. | n/a |
| Results and Discussion: - This article points out the need for accessible instruments for microscopy; however, it is briefly discussed about implications of national regulation for medical devices. Can an improved prototype be used in health facilities? Please, provide more context or information about this topic. | Dear Reviewer,  Thank you for this comment.  We believe an improved prototype could be used in health facilities but that there are more stages in its development needed to be done to be confident on its performance in health facilities.  Using the [technology readiness levels (TRL) framework](https://www.twi-global.com/technical-knowledge/faqs/technology-readiness-levels), the OFM we produced would classify as TRL 4, where we have validated its performance in viewing slide images not yet in a simulated or operational environment.  We are currently undertaking a separate project where we are redesigning the OFM based on feedback by different end-users including medical technologists, medical students, pathologists, and primary care physicians so that it would be more readily be usable in their operations. The results of this project we believe would effectively push our prototype to TRL 6 having demonstrated it to end-users in a simulated environment.  We then plan to deploy our redesigned OFM to an underserved rural facility, evaluate its performance, and iterate as necessary to achieve reliable operation in these contexts including a realistic assessment of its diagnostic performance. If this is successful, we would be able to push the redesigned OFM to TRL 7 or 8. By this point given our demonstrated ability to do distributed manufacturing we could, in theory, continue to make these microscopes depending on need and customize as necessary based on further inputs from the field.  Transitioning to TRL 9, a fully commercial product has its own challenges. Philippine Medical device regulations may already be applicable as the intent is commercialization of the redesigned OFM. Our view is that microscopes by themselves are not covered under the legal definition of a medical device in the Philippines according to the FDA act of 2009, as it is more of a general laboratory instrument used in many contexts like general education, research, and in industry for viewing microscopic features of samples. Within a healthcare service context, microscopes are tools that allow trained personnel to view microscopic images from prepared samples.  While it could be argued that the OFM could fit into the definition of a medical device, this is a subject of discussion with regulators and other stakeholders once our team decides to fully commercial the product.  Our text on future directions touches upon these plans already. We believe that by the time we can achieve TRL 7 or 8 that we would be confident to place it in health facilities if leaders are willing to adopt the technology at this stage of development.  A caveat to this is that because the OFM is an open-source design, it is completely possible that health facilities with some engineering know-how (or partners that could do this for them) and resources to buy the parts, 3d printer, and modelling software could fabricate and assemble their own OFM and develop the capacity to implement them in regular operations independently.  Having said these, we have stated in the limitations discussion that the current design is not usable in health facilities based on our assessment and we have discussed future work that could maximize its chances of use, adoption, and implementation by health facilities. | Please see discussion section, lines 393 to 428, pages 24 to 27  Please see conclusion section lines 476 to 483, page 29 |
| Conclusions - It cannot be concluded that the OFM is better than the mobile phones when only one model was used in this study. The quality and performance will depend on the camera sensor and lenses. | Dear reviewer  Thank you for this comment. We have removed text stating that our OFM is better than mobile phones used with conventional microscopes. | Please see conclusion section lines 430 to 435, page 27 |
| - Please, describe which kind of objective you used. For 100x, there are oil and dry objectives. | Dear reviewer,  We have used a 100x oil immersion objective. We also stated that we were able to focus still without oil because of software correction techniques | Please see table 6 row 2. |
| - Please, give more information about protocols to be adapted. Standard sample preparation protocols can be used with the OFM. User guides may be different due to the technology. | Dear reviewer,  We have already stated some of the protocols and intended diagnostic procedures being targeted for adoption with the OFM in our text. | Please see introduction section lines 34 to 37, page 2  Please also see conclusion section 465 to 468, page 28 |

We hope our responses are satisfactory and we are happy to provide more clarifications and responses with the contents of our paper.

Very sincerely yours,

Jeremie E. De Guzman MD., MBA., MSc
